# Management of Saddle Pulmonary Embolism in the Context of Heparin-Induced Thrombocytopenia and Granulomatosis With Polyangiitis

**DOI:** 10.7759/cureus.72096

**Published:** 2024-10-22

**Authors:** Doaa Subahi, Huria Huma, Mohammad Faize, Wadah Ibrahim

**Affiliations:** 1 Respiratory Medicine, Glenfield Hospital, University Hospitals of Leicester NHS Trust, Leicester, GBR; 2 Respiratory Medicine, University of Leicester, Leicester, GBR

**Keywords:** argatroban, granulomatosis with polyangiitis, heparin-induced thrombocytopenia, pulmonary embolism, warfarin

## Abstract

Heparin-induced thrombocytopenia (HIT) is a serious, immune-mediated complication of heparin therapy that paradoxically increases the risk of thrombosis while causing a significant reduction in platelet count. In this report, we present the case of a 65-year-old Caucasian female with a history of granulomatosis with polyangiitis (GPA) and acute kidney injury requiring dialysis who was admitted with progressive shortness of breath. Imaging confirmed the presence of a saddle pulmonary embolus and multiple segmental emboli, complicated by bilateral pulmonary infarcts. Given her history of HIT, direct thrombin inhibitors (DTIs) were initiated, with argatroban used in conjunction with warfarin. Close monitoring of anticoagulation was performed using activated partial thromboplastin time (APTT) and international normalized ratio (INR) trends. The case highlights the challenges of managing pulmonary embolism (PE) in the context of HIT and GPA, emphasizing the need for multidisciplinary decision-making and individualized anticoagulation strategies.

## Introduction

Pulmonary embolism (PE) is a life-threatening condition that is frequently treated with anticoagulation, often involving heparin. However, heparin-induced thrombocytopenia (HIT) is an immune-mediated reaction to heparin, resulting in significant thrombocytopenia and an elevated risk of thrombosis. This paradoxical response leads to clot formation rather than bleeding, complicating the management of PE by increasing thrombotic risk despite low platelet counts. The incidence of thrombosis in confirmed HIT can be as high as 50% at presentation, often occurring before a detectable decline in platelet count [1]. Heparin-induced thrombocytopenia has a mortality rate of 5% to 10%, with substantial morbidity occurring in 30% of patients when diagnosis or treatment is delayed [1]. Thrombosis can present before any significant drop in platelet count, further complicating early detection. 

Typical anticoagulation therapy, using agents like unfractionated heparin (UFH) and low molecular weight heparin (LMWH), prevents thrombus formation by inhibiting thrombin and factor Xa, thereby reducing clotting activity without significantly activating platelets. In contrast, HIT leads to an increased risk of thrombosis due to platelet activation by antibodies, resulting in thrombocytopenia and thrombotic events. Management of acute PE in the context of HIT requires careful selection of alternative anticoagulants, as traditional UFH is contraindicated. Direct thrombin inhibitors (DTIs), such as argatroban and bivalirudin, are recommended, particularly in cases where HIT is confirmed. Ultimately, HIT transforms a therapeutic anticoagulant into a catalyst for serious thrombotic complications.

Granulomatosis with polyangiitis (GPA) is a small-vessel vasculitis that complicates the management of HIT, as both involve immune-mediated processes. It causes vascular inflammation and potential thrombocytopenia, while HIT increases thrombosis risk through platelet activation. This necessitates careful management and often the use of alternative anticoagulants to prevent complications. This report outlines the management of a complex case involving a saddle PE in a patient with a history of HIT and GPA, focusing on the use of alternative anticoagulation strategies and the importance of multidisciplinary collaboration in achieving a favorable outcome.

## Case presentation

A 65-year-old Caucasian woman, initially admitted in January 2024 for pneumonia, faced a series of severe complications that unfolded during her hospital stay. Upon admission, she was found to have acute kidney injury, with her estimated glomerular filtration rate (eGFR) dropping from a baseline of 73 to a 7. Concurrently, her urea levels increased from 6.9 to 32.0 and creatinine levels from 75 to 505, for which she was commenced on dialysis.

Further investigations revealed a normal urinary tract ultrasound, but a vasculitis screen returned a positive result for PR3 antineutrophil cytoplasmic antibodies (ANCA) at 106.9, and she was started on plasma exchange treatment alongside IV cyclophosphamide and methylprednisolone. A CT scan of her thorax showed multifocal pulmonary hemorrhage, raising suspicion of pulmonary-renal syndrome.

Amidst these challenges, she also developed thrombocytopenia, with her platelet count dropping to 60. A HIT screen returned positive (12.8), prompting a switch from prophylactic dalteparin to an argatroban infusion, alongside the initiation of bridging therapy with warfarin. She was advised to continue warfarin for four weeks post-discharge, extending into February 2024.

Upon her return to the hospital in April 2024, she presented with a week's history of worsening shortness of breath, requiring 1 L of supplemental oxygen. Her vital signs showed a blood pressure of 115/75 and a heart rate of 99 bpm. Her medication history included calcium acetate (950 mg twice daily), long-term co-trimoxazole (480 mg daily for *Pneumocystis carinii *pneumonia (PCP) prophylaxis), and a weaning dose of prednisolone and cyclophosphamide, which was transitioned to azathioprine after thiopurine methyltransferase (TPMT) levels were found just below the normal range (64). Notably, she was also on roxadustat (70 mg, three times a week), which was considered the triggering cause of the PE, given that venous thromboembolism is a known side effect of the medication.

**Figure 1:**
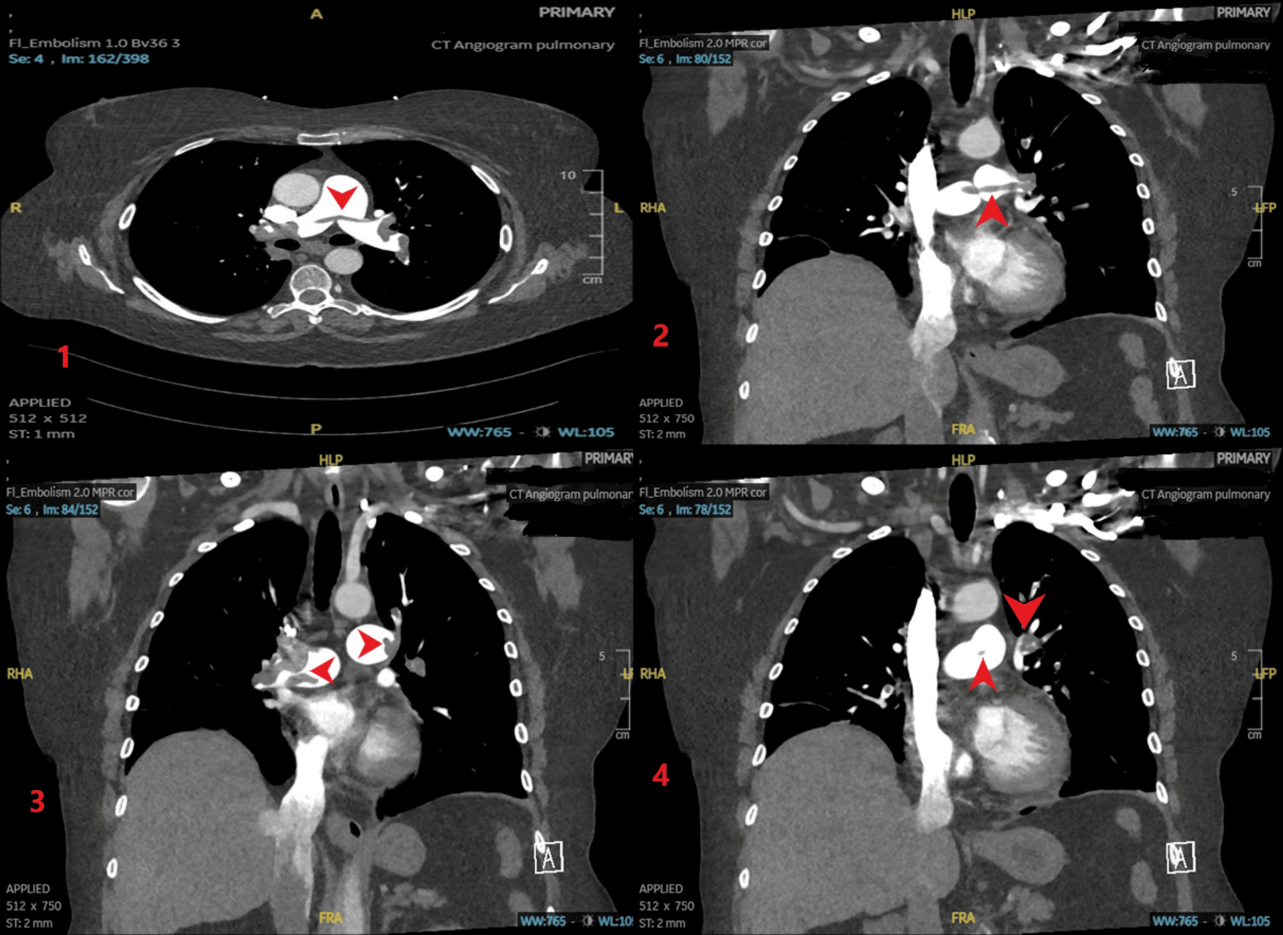
The CTPA showing saddle and bilateral segmental PE, and evidence of pulmonary hypertension and right heart strain. CTPA: Computed tomography pulmonary angiography, PE: Pulmonary embolism

Initial blood investigations revealed a hemoglobin level of 104, which remained stable throughout her admission, while her platelet count was within normal limits at 265. Troponin levels were 1282.3 and D-dimer was 9. Her most recent brain natriuretic peptide (BNP) was 571 (March 2024), and her pulmonary embolism severity index (PESI) score was 65 points (primarily influenced by her age), which classified her in the European Society of Cardiology (ESC) moderate-risk group with a systematic coronary risk evaluation 2 (SCORE2) of 4.4%. A CT pulmonary angiography (CTPA) confirmed a bilateral central and segmental PE complicated by a saddle embolus and bilateral pulmonary infarcts (Figure 1).

**Figure 2:**
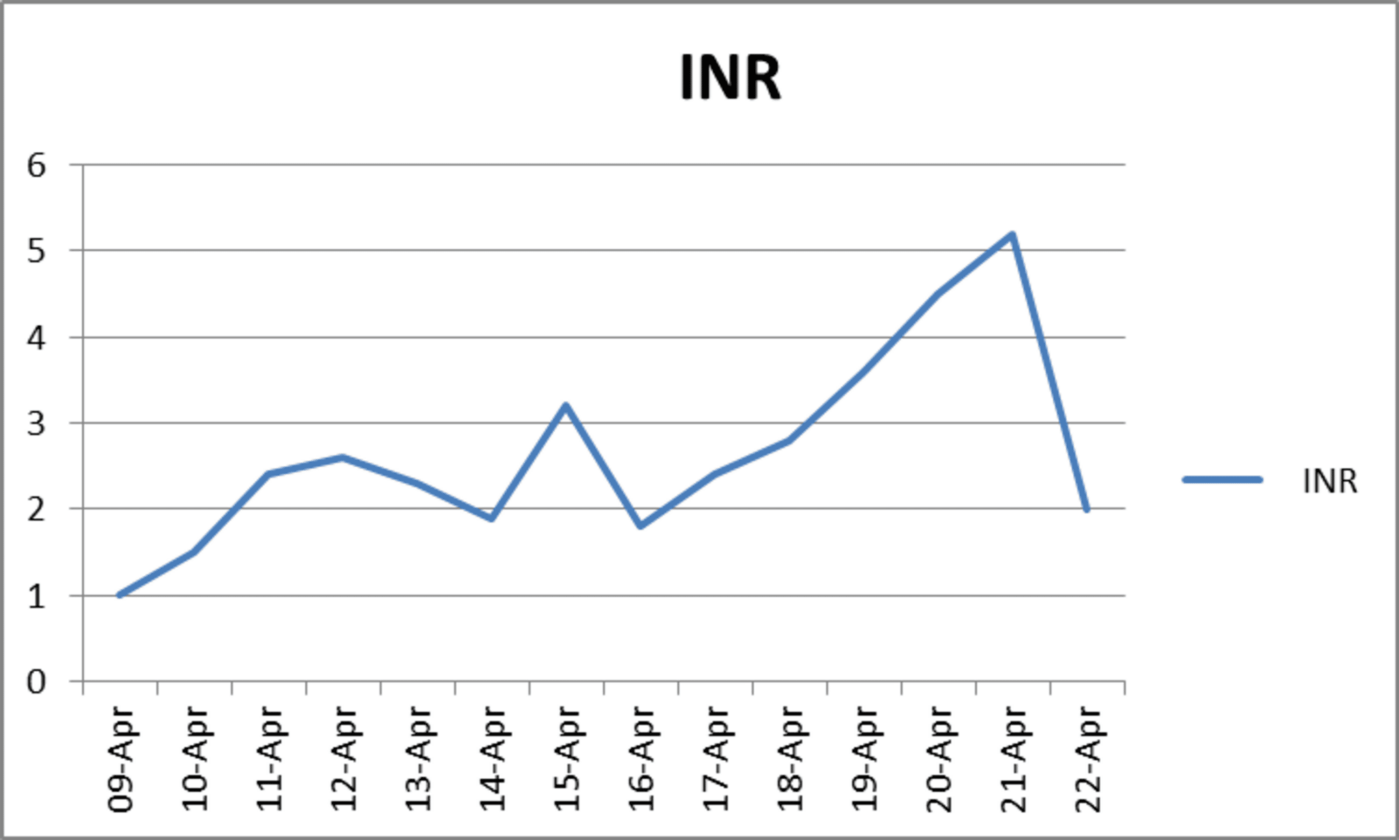
The patient's INR trend The figure displays the INR trend over the course of treatment. The target INR was set at 4, with the highest recorded INR value being 5.2 and the lowest being 1.0. The mean INR throughout the treatment period was 2.7. INR: International normalized ratio

**Figure 3:**
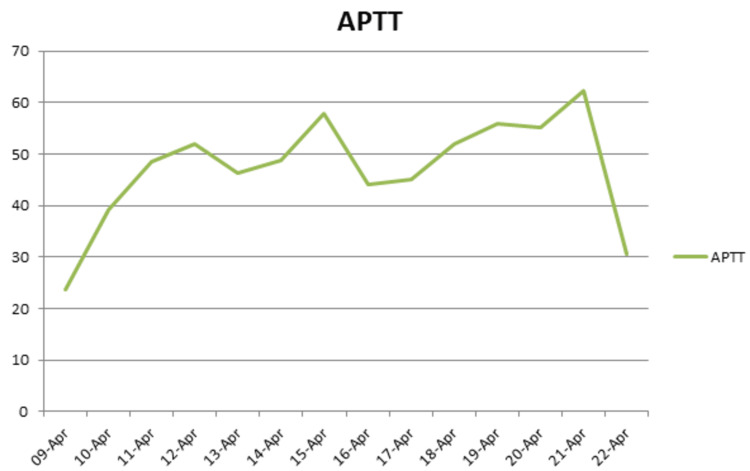
The patient's APTT trend This figure illustrates the APTT trend during treatment. The target APTT range was 1.5 to 3 times the control. The highest recorded APTT value was 57.0, while the lowest was 23.7, with a mean APTT of 47.3. APTT: Activated partial thromboplastin time

In light of her chronic kidney disease, her initial serum creatinine was elevated at 263, with urea levels at 15 and an eGFR of 16. With a slight improvement during her admission: creatinine level dropped to 206, urea was at 10.7, and eGFR was at 21. Given her history of HIT, a multidisciplinary team including hematology and intensive care specialists was consulted. Argatroban, a DTI, was initiated per local guidelines due to its safety profile in patients with HIT and its renal metabolism, making it a suitable choice given her chronic kidney disease. The international normalized ratio (INR) and activated partial thromboplastin time (APTT) were monitored closely to titrate Argatroban (Figures 2-3). 

Upon admission, the patient was commenced on an argatroban infusion for anticoagulation management. Five days later, following significant clinical improvement, the patient was weaned off oxygen therapy, allowing for the initiation of bridging therapy with warfarin. After six days of daily INR monitoring, the INR rose to 4.5, prompting adherence to the trust's guidelines, which recommend discontinuing argatroban in such circumstances and transitioning to warfarin monotherapy. The patient exhibited a positive response to this treatment strategy, with a marked improvement in shortness of breath. A follow-up echocardiogram performed two months post-discharge revealed a nondilated right heart, normal right ventricular (RV) function, and no evidence of pulmonary hypertension. 

## Discussion

Heparin-induced thrombocytopenia is a prothrombotic adverse drug reaction that occurs in less than 0.1% to 7% of patients treated with heparin, depending on the specific patient population and the type of heparin administered [2]. The management of HIT is significantly dependent upon a series of diagnostic tests, which may not be available when timely clinical decisions are required [2]. 

Patients undergoing cardiac surgery are at a higher risk for HIT compared to the general hospital population. The risk of HIT is influenced by the type of heparin administered. Low molecular weight heparin is associated with a lower incidence of HIT compared to UFH. This is because LMWH forms ultra-large complexes with platelet factor 4 (PF4) less efficiently than UFH, resulting in fewer heparin-induced antibodies [3]. Specifically, the incidence of HIT with UFH is approximately 22 per 1,000 patients, whereas for LMWH it is around five per 1,000 [3]. Furthermore, HIT complicated by venous thromboembolism occurs significantly less frequently in patients receiving LMWH (four per 1,000) compared to UFH (17 per 1,000) [3]. 

In response to these findings, some institutions have implemented quality improvement initiatives such as the ‘Avoid-Heparin Initiative,’ which replaces UFH with LMWH. This approach has led to a notable 79% reduction in HIT incidence [3]. For stable patients without severe renal or hepatic impairment and who are not at risk of bleeding, alternative non-heparin anticoagulants can be used. Fondaparinux and direct oral anticoagulants (DOACs) are often recommended as first-line alternatives following danaparoid or argatroban [4]. For unstable patients, those at risk of bleeding, or individuals in intensive care units, short-acting injectable anticoagulants such as argatroban or bivalirudin are preferred, accompanied by rigorous biological monitoring. In severe cases of HIT, such as massive PE, extensive arterial thrombosis, or venous gangrene, argatroban or bivalirudin are used as primary treatments, also with strict monitoring [4]. Patients with severe renal impairment (creatinine clearance < 30 mL/min) should only receive argatroban. In those with severe hepatic impairment (Child-Pugh class C), bivalirudin, danaparoid, or fondaparinux can be used [4]. 

Vasculitis and HIT originate from different underlying causes, yet both involve autoimmune processes that complicate clinical management. Heparin-induced thrombocytopenia is associated with the presence of antibodies that recognize complexes of PF4 and heparin [5]. Development of ANCA in ANCA-associated vasculitis is linked with the loss of immunological T-cell and B-cell tolerance to one of two neutrophil proteins: leukocyte proteinase 3 or myeloperoxidase [5]. Treating vasculitis often involves anticoagulants like heparin, which can inadvertently cause HIT, leading to serious thrombotic complications. Retrospective observational studies show significant links between HIT and conditions such as hemodialysis, autoimmune diseases, gout, and heart failure, highlighting the complex risks for these patients [6]. In hemodialysis, regular heparin use increases HIT risk, with a notable Japanese study reporting an incidence of 3.9% [6].

Both vasculitis and HIT increase the risk of thrombosis and bleeding complications. A study found clinically significant bleeding in 41% of HIT cases [5]. In vasculitis, pulmonary hemorrhage can be life-threatening, and bleeding in other systems has been documented as an initial sign of the disease [5]. The overlap in symptoms can lead to misdiagnosis and inappropriate treatment, worsening patient outcomes. It's vital to consider the potential co-occurrence of these conditions, as patients with new-onset vasculitis often receive heparin early for thromboprophylaxis or during dialysis.

The management of PE in patients with HIT and GPA requires a tailored approach due to the complexities associated with these conditions. Heparin-induced thrombocytopenia significantly increases the risk of thrombosis, necessitating the use of alternative anticoagulants such as direct thrombin inhibitors. As per our local hematology guidelines, argatroban is effective for managing PE without exacerbating thrombocytopenia. Bridging DTIs with warfarin is critical for achieving and maintaining therapeutic INR targets. This process involves daily monitoring of INR and APTT ratios to adjust dosages of both argatroban and warfarin, minimizing the risks of over- or under-anticoagulation. Once the target INR and APTT ratios are achieved, argatroban is discontinued, and warfarin therapy is continued with regular INR monitoring to ensure sustained anticoagulation. Patient education is crucial, involving the provision of a warfarin booklet, an alert card, and information on the importance of regular INR checks, potential side effects, and the new target INR range of 2 to 3 to ensure effective long-term management. 

## Conclusions

This case underscores the complexities of managing PE in the context of HIT and GPA. The successful use of argatroban, followed by a careful transition to warfarin, highlights the importance of alternative anticoagulation strategies when traditional options like heparin are contraindicated. Multidisciplinary care, close monitoring, and patient education were key factors in achieving a positive outcome in this high-risk patient. This case adds to the clinical experience supporting the use of DTIs in patients with HIT and background ANCA-positive vasculitis (GPA) and provides valuable insights for clinicians managing similar cases. 
